# Palliative care in CADASIL: diagnosis is only the first step

**DOI:** 10.1055/s-0043-1777009

**Published:** 2023-11-30

**Authors:** Victor Aguilar-Fuentes, Diego Justo-Hernández, José Miguel Arredondo-Dubois, José Luis Ruiz-Sandoval, Amado Jiménez-Ruiz

**Affiliations:** 1 Benemérita Universidad Autónoma de Puebla, Facultad de Medicina, Puebla de Zaragoza, Puebla, México.; 2 Universidad de Guadalajara, Facultad de Medicina, Guadalajara, Jalisco, México.; 3 Antiguo Hospital Civil de Guadalajara, Guadalajara, Jalisco, México.; 4 Hospital Civil de Guadalajara “Fray Antonio Alcalde”, Departamento de Neurología, Clínica de Enfermedad Vascular Cerebral, Guadalajara, Jalisco, México.

Dear Editor,

We read with interest the article published by Nogueira et al. 1 titled “Clinical and epidemiological profiles from a case series of 26 Brazilian CADASIL patients.” We congratulate the authors for their very descriptive case series.

We noticed the authors did not actively recruit family members from affected individuals. Nevertheless, we consider family counseling to be an essential factor in patients diagnosed with cerebral autosomal dominant arteriopathy with subcortical infarcts and leukoencephalopathy (CADASIL). We would like to propose an alternative approach using the following case report we recently diagnosed.

**Figure 1:**
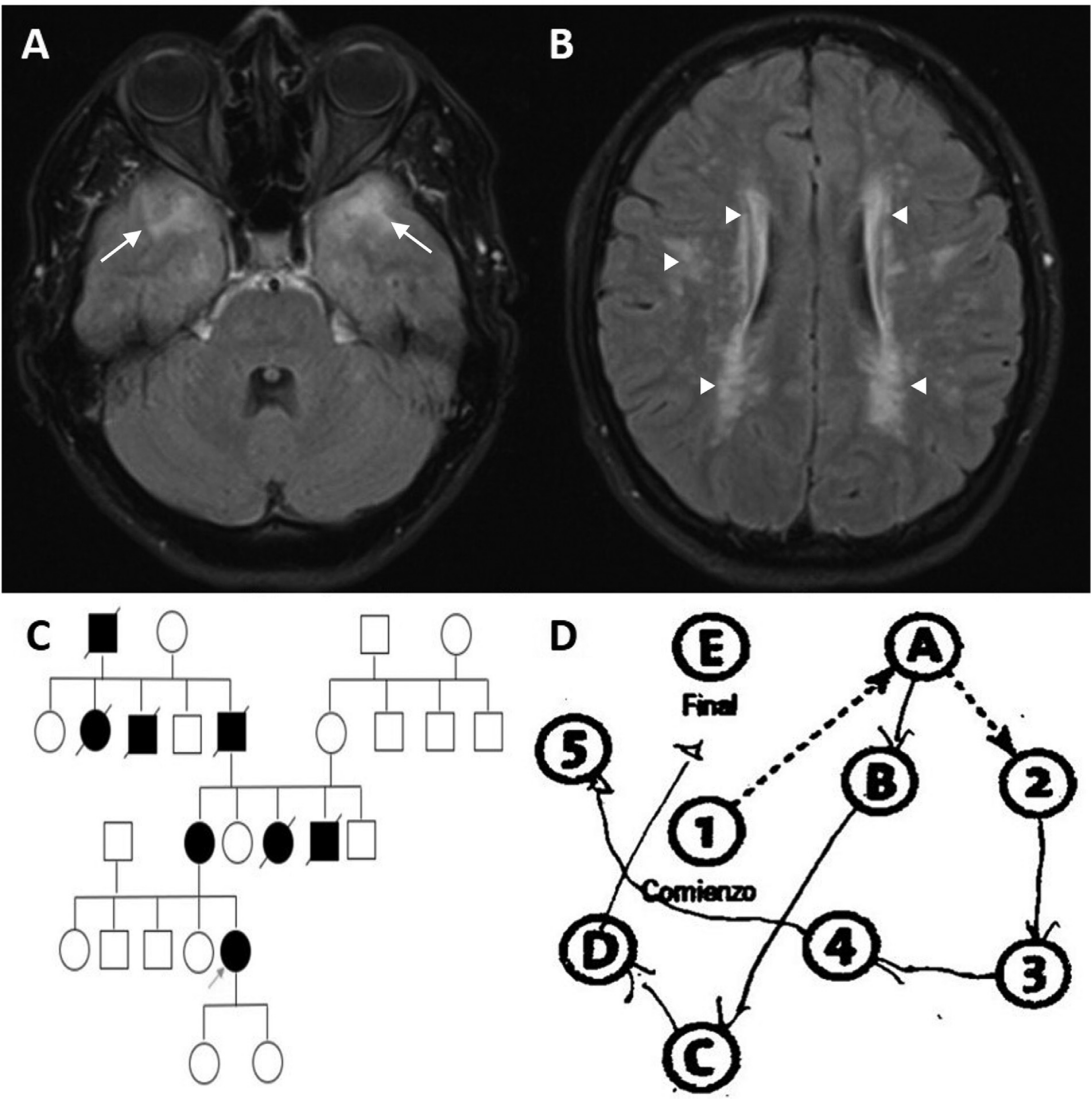
( **A,B** ) Fluid-attenuated inversion recovery (FLAIR) MRI scan showing extensive bilateral supratentorial leukoencephalopathy with characteristic involvement of the anterior temporal lobes (arrows) and periventricular and subcortical white matter hyperintensities (arrowheads). The cerebral cortex is not involved. ( **C** ) Family tree showing an autosomal dominant disease pattern. Black squares and circles represent affected relatives. An arrow indicates the patient. ( **D** ) Trail making test revealing profound visuospatial/executive dysfunction.

We initially suspected CADASIL in a 66-year-old female patient who presented with cognitive impairment and stroke to the neurology department. After diagnostic confirmation using genetic testing, we evaluated the medical records of the relatives and found six family members with a history of cognitive impairment and death from ischemic stroke at an early age ( Figure 1C ). We evaluated the patients' daughter, who presented with a 6-month history of depression, headaches, forgetfulness, and inattention. Physical examination was unremarkable. We performed a Montreal Cognitive Assessment (MoCA) test, which showed visuospatial/executive multidomain impairment (19/30 points). ( Figure 1D )

Magnetic resonance imaging (MRI) demonstrated white matter hyperintensities in the anterior temporal lobes and deep subcortical white matter ( Figure 1A,B ). Genetic testing was positive for *NOTCH3* c.437G > A (p.Cys146Tyr) mutation confirming the diagnosis of CADASIL.

We started the patient on a selective serotonin reuptake inhibitor (SSRI) and aspirin and referred her to medical genetics for further workup. After a 5-week follow-up, the patient reported significant mood improvement. Although a modest intervention, we believe it positively impacted her quality of life in this challenging situation.

CADASIL is a rare genetic disease. Clinically it presents with headache, mood disturbances, apathy, cognitive impairment, and subcortical small vessel disease. 2 3 This entity must always be considered in patients with a family history of cognitive impairment and early-onset stroke.

CADASIL has an autosomal dominant inheritance. Hence, genetic counseling of the patient's relatives can aid in early detection of mood, behavioral, and cognitive disturbances. The precise cutoff for the initial evaluation of the relatives is unclear. However, it must be performed as early as possible. Mood and behavioral disturbances present in up to 50% of patients with CADASIL and can have detrimental effects on the patients' lifestyle, including withdrawal from work, violence, and suicidal ideation. Early detection of these disturbances results in a promptly therapeutic approach with significant improvement in the patient's and family's well-being. 4

As neurologists, we must be acquainted with the symptomatic treatment of patients to ensure a good quality of life, even in progressive and irreversible diseases such as CADASIL. Radiological findings may also help in the initial evaluation of the patient. Imaging studies have found a positive correlation between white matter lesions (such as those exemplified in this case) and depressive symptoms. 4

Mood improvements may substantially affect the social interaction of the patient. Ensuring mental health increases the quality of life of the patient and that of the relatives, relieving caregiver burden.

Treatment of CADASIL is usually supportive, with no available disease-modifying drugs. 5 Even in this context, we believe it is necessary to offer family screening to identify affected subjects and recognize early symptoms that may benefit from early intervention.

Quality of life embodies overall well-being, happiness, and physical and emotional health. As physicians, we must never forget this powerful intervention:

*“To cure sometimes, to relieve often, to comfort always.”*
